# FGT‐1‐mediated glucose uptake is defective in insulin/IGF‐like signaling mutants in *Caenorhabditis elegans*

**DOI:** 10.1002/2211-5463.12068

**Published:** 2016-04-21

**Authors:** Shun Kitaoka, Anthony D. Morielli, Feng‐Qi Zhao

**Affiliations:** ^1^ Laboratory of Lactation and Metabolic PhysiologyDepartment of Animal and Veterinary SciencesUniversity of VermontBurlingtonVTUSA; ^2^ Department of PharmacologyCollege of MedicineUniversity of VermontBurlingtonVTUSA; ^3^ Present address: Drug Discovery LaboratoryWakunaga Pharmaceutical Co., Ltd.AkitakataHiroshimaJapan

**Keywords:** *Caenorhabditis elegans*, glucose transporter, insulin/IGF‐like signaling, post‐transcriptional regulation

## Abstract

Insulin signaling plays a central role in the regulation of facilitative glucose transporters (GLUTs) in humans. To establish *Caenorhabditis elegans* (*C. elegans*) as a model to study the mechanism underlying insulin regulation of GLUT, we identified that FGT‐1 is most likely the only functional GLUT homolog in *C. elegans* and is ubiquitously expressed. The FGT‐1‐mediated glucose uptake was almost completely defective in insulin/IGF‐like signaling (IIS) mutants *daf‐2* and *age‐1*, and this defect mainly resulted from the down‐regulated FGT‐1 protein expression. However, glycosylation may also be involved because OGA‐1, an *O*‐GlcNAcase, was essential for the function of FGT‐1. Thus, our study showed that *C. elegans* can be a new powerful model system to study insulin regulation of GLUT.

**Abbreviations**
2DG: 2‐deoxy‐d‐glucose
^3^H‐2DG: tritium‐labeled 2DG
*C. elegans*: *Caenorhabditis elegans*
FGT: facilitative glucose transporter in *C. elegans*
GLUT: facilitative glucose transporter
IGF: insulin‐like growth factor
IIS: insulin/IGF‐like signaling
SGLT: Na^+^/glucose cotransporter
wt: wild‐type

Insulin is a central regulator for sustaining glucose homeostasis in humans. When blood glucose levels increase after meals, insulin is secreted to activate glucose uptake and utilization in peripheral tissues, mainly muscle and adipose tissues 1. This stimulation is primarily mediated by the insulin‐induced subcellular translocation of the insulin‐sensitive facilitative glucose transporter (GLUT) 4. In patients with type II diabetes, the regulation of GLUT4 by insulin is defective, and muscle and fat cells become unresponsive to insulin signals, which is termed as insulin resistance 2, 3. The mechanisms underlying insulin resistance still remain largely unknown.

Insulin signaling is highly conserved in the animal kingdom, including *Caenorhabditis elegans* (*C. elegans*). In *C. elegans*, most of the major signaling molecules in the insulin cascade are well conserved, although insulin‐like growth factors (IGF) may function through the same cascade [termed insulin/IGF‐like signaling (IIS)] unlike in humans, and IIS has been known to play important functions in many physiological processes, such as reproduction, growth, metabolism, and aging 4, 5, 6, 7, 8. For instance, knockdown of DAF‐2, the only homolog of the mammalian insulin/IGF‐1 receptor, dramatically increases the lifespan of *C. elegans* 5, 9, 10. This lifespan extension is dependent on DAF‐16, the homolog of mammalian FOXO transcription factor 10. Importantly, the presence of glucose impairs lifespan extension of IIS mutants by downregulating DAF‐16 11. Because of its advantage of relatively easy genetic manipulation and the availability of a large collection of mutant strains, in addition to its many other unique advantages for study, such as a short lifespan and quick turnover, *C. elegans* may be an attractive model system to study the mechanisms underlying insulin regulation of glucose transport.

To explore *C. elegans* as a model system for the study of insulin regulation of GLUT functions, we characterized nine GLUT candidates in *C. elegans* (FGT) based on their high sequence homologies to human GLUTs in our previous study 12. FGT‐1 was identified as the sole counterpart of GLUT with the ability to transport glucose in *Xenopus* oocytes. The localization of FGT‐1 was mainly restricted to the digestive tract when expressed under its 2 kb promoter sequence 12, suggesting that there are other unidentified FGT isoforms in other tissues or that the 2 kb FGT‐1 promoter sequence cannot faithfully represent its endogenous promoter function. However, our previous findings were not able to determine whether FGT‐1 is regulated by IIS in *C. elegans*.

In this study, we investigated the tissue localization of another FGT‐1 alternative splicing isoform, FGT‐1B, and re‐examined the tissue localization of FGT‐1A in response to a longer promoter sequence. We also showed that FGT‐1 is regulated by IIS by comparing the differences in glucose uptake between FGT‐1 mutants in wild‐type (wt) or IIS mutant backgrounds. We further investigated the regulatory mechanisms of IIS on FGT‐1 function.

## Materials and methods

### Plasmid constructions and *C. elegans* strains and culture

All plasmids and *C. elegans* strains used in this study are described in Data S1. The *C. elegans* strains were cultivated at 20 °C under standard conditions unless otherwise specified 13.

### Glucose transport assay in *Xenopus* oocytes

The cRNAs of *fgt‐1a* were generated in our previous study 12. cRNAs of *fgt‐1b*,* fgt‐1a(tm3165),* and *fgt‐1b(tm3165)* were synthesized by *in vitro* transcription from pSP‐fgt1b, pSP‐fgt1atm, and pSP‐fgt1btm, respectively, using the mMessage mMachine kit (Ambion, Austin, TX, USA). The 2DG uptake analysis of wt or mutated FGT‐1A and FGT‐1B was performed in *Xenopus* oocytes as described previously 12, 14.

### Glucose uptake assay of intact worms

Synchronized young adult worms were washed out from the culture plates and incubated in M9 saline for 1 h. A 10% volume of worms was collected for protein quantitation. The remaining worms were subjected to the uptake assay using 0.5 mm 2DG containing 3 μCi ^3^H‐2DG in M9 saline in the presence or absence of 100 μm phloretin or phlorizin. The worms were incubated in the uptake solutions for 2 h at 20 °C and then washed thoroughly three times with ice‐cold PBS containing 0.5% Tween‐20 prior to lysis in 0.5% SDS containing 60 μg·mL^−1^ proteinase K for 1 h at 55 °C. The radioactivity of the lysed worms was counted using a Tri‐Carb Liquid Scintillation Counter 2900TR (Perkin Elmer Inc., Waltham, MA, USA). Each experiment was performed independently four times.

### Feeding RNAi

RNAi feeder plasmids of DAF‐2 and DAF‐16 were obtained from Addgene (Cambridge, MA, USA; plasmid #34833 and #34834). An RNAi feeder plasmid of AGE‐1 was obtained from Source Bioscience (Kennesaw, GA, USA). RNAi feeder plasmids of AKT‐1 and OGA‐1 were obtained from GE Healthcare Dharmacon Inc (Pittsburgh, PA, USA). These plasmids were transformed into HT115 (DE3) bacteria, and RNAi was performed by culturing the worms on plates together with these feeding bacteria.

### mRNA quantitation and western blot analysis

The mRNA levels of FGT‐1, DAF‐2, AGE‐1, AKT‐1, DAF‐16, and OGA‐1 were measured by reverse transcription quantitative PCR (RT‐qPCR) with primer pairs FGT1q, DAF2q, AGE1q, AKT1q, DAF16q, and OGA1q, respectively (Table S1) 15. The mRNA levels of CDC‐42 and PMP‐3, which were analyzed with primer sets CDC42q and PMP3q, respectively (Table S1), served as internal controls to normalize the expression of the other mRNA 16. RT‐qPCR and western blot analysis were performed as described previously 12. Band intensity of the western blot was quantified with image lab 4.1 (BioRad Laboratory, Hercules, CA, USA) and normalized to the level of β‐actin.

### Statistical analysis

For the 2DG transport assay in oocytes, any uptake in *fgt‐1a* or *fgt‐1b* cRNA‐injected oocytes less than three times the mean value of water‐injected oocytes was considered as injection failure and discarded from the analysis. The 2DG uptake in *fgt‐1a* or *fgt‐1b* cRNA‐injected oocytes was corrected by subtraction of the mean 2DG uptake of water‐injected oocytes.

The statistical analysis of the individual experiments is indicated in the figure legends, and the analyses were conducted using graphpad prism 6.03 (GraphPad Software, La Jolla, CA, USA) and jmp pro 11.2 (SAS Institute Inc., Cary, NC, USA).

## Results

### Tissue localization and glucose transport activity of FGT‐1A and ‐1B

**Figure 1:**
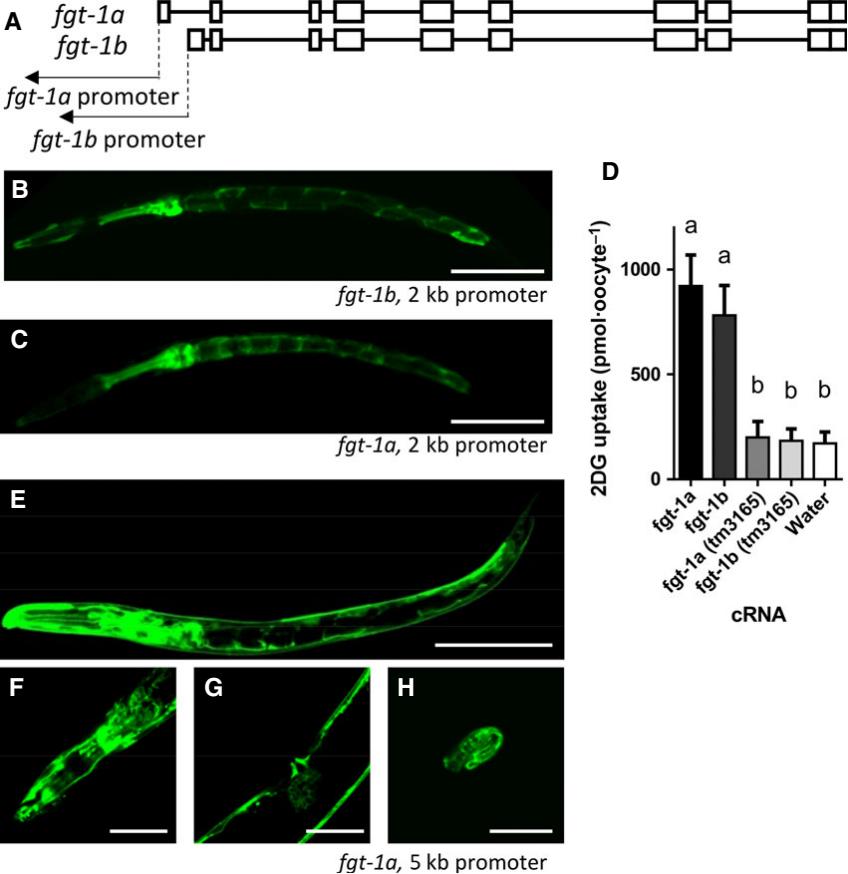
Tissue localization and glucose transport activity of the FGT‐1 isoforms A and B. (A) Gene structures of *fgt‐1a* and *fgt‐1b*. The boxes represent exons, and the lines connecting the boxes represent introns. (B, C) Tissue localization of FGT‐1B::GFP (B) and FGT‐1A::GFP (C) in L2 worms, driven by the corresponding 2 kb upstream promoter sequences of *fgt‐1a* and *fgt‐1b*. (D) 2‐deoxy‐d‐glucose (2DG) uptake activity of wild‐type FGT‐1A and ‐1B or mutated FGT‐1A and ‐1B which were cloned from *fgt‐1(tm3165)* mutant animals in *Xenopus* oocytes injected with the corresponding wild‐type or mutated FGT‐1A and ‐1B cRNA or water. Data are represented as means with SD from three independent experiments with 10 or more oocytes for each analyses. The statistical analysis was conducted using one‐way ANOVA with Tukey's HSD test, and significant differences are indicated by the different alphabetical letters on each bar (*P* < 0.001). (E–H) Tissue localization of the FGT‐1A::GFP fusion protein in L2 worms, driven by the 5 kb upstream promoter sequence of *fgt‐1a*. GFP images of the whole body at the L2 stage (E), head (F) and midgut (G) of the young adult, and embryo (H). Scale bars indicate 50 μm.

Because FGT‐1(A) was identified as the sole GLUT homolog in *C. elegans* with glucose transport activity and its expression was mainly observed in the digestive tract in our previous study 12 *,* we hypothesized that another FGT‐1‐splicing isoform, FGT‐1B, is expressed in other tissues. FGT‐1B utilizes a distinct exon 1 from FGT‐1A and, therefore, has a slightly different promoter sequence (186 bp of extra sequence at the 3′ end, Fig. 1A). To determine whether FGT‐1A and ‐1B have isoform‐specific tissue localizations, we expressed FGT‐1A::GFP and FGT‐1B::GFP under the corresponding 2 kb upstream promoter sequences of *fgt‐1a* and *fgt‐1b* in wt *C. elegans*. FGT‐1B showed the same localization as FGT‐1A: primarily in the pharynx and intestinal cells (Fig. 1B,C). The glucose transport activity of FGT‐1B was also compared with FGT‐1A in *Xenopus laevis* oocytes (Fig. 1D). The uptake activity of FGT‐1B for 2‐deoxy‐d‐glucose (2DG) did not differ from that of FGT‐1A as reported previously 17, which indicated that the small structural difference between FGT‐1A and FGT‐1B at the N‐terminus does not alter sugar transport activity.

To examine whether the 2 kb upstream sequence of FGT‐1 faithfully represents the endogenous localization of FGT‐1, we reanalyzed the tissue localization of FGT‐1A::GFP driven by a longer 5 kb promoter sequence. As shown in Fig. 1E–H, The 5 kb promoter drove FGT‐1A::GFP expression not only in the digestive tract like the 2 kb promoter but also throughout the body, including in the head neurons, vulva, and body wall muscle. This observation suggests that FGT‐1 may be ubiquitously expressed in *C. elegans*, and that the 2 kb promoter sequence may not be long enough to include all of the regulatory elements of the *fgt‐1* promoter.

### The IIS mutant has impaired facilitated glucose transport activity

**Figure 2:**
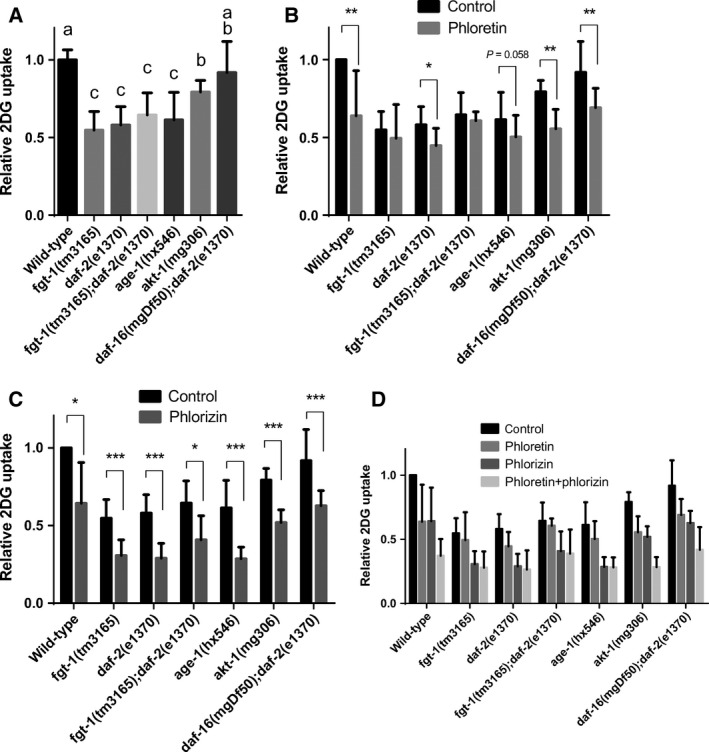
Glucose uptake activity in *fgt‐1(tm3165)* and insulin/IGF‐like signaling in mutant worms. (A) Intact worms of wild‐type and mutants of *fgt‐1(tm3165), daf‐2(e1370), fgt‐1(tm3165); daf‐2(e1370), age‐1(hx546), akt‐1(mg306),* and *daf‐16(mgDf50); daf‐2(e1370)* were incubated with tritium‐labeled 2‐deoxy‐d‐glucose (^3^H‐2DG) for 2 h and evaluated for 2DG uptake. Uptake in the wild‐type worm was set at 1. Error bars represent the SD (*N* = 4). The statistical analysis was conducted using one‐way ANOVA with Tukey's HSD test, and bars not sharing a common letter are significantly different (*P* < 0.05). (B–D) Intact worms of wild‐type and mutants were incubated with ^3^H‐2DG in the presence or absence of phloretin (B), phlorizin (C), or both inhibitors (D) for 2 h and evaluated for 2DG uptake. Uptake in wild‐type worms in the absence of any inhibitors was set at 1. Error bars represent SD in four independent experiments. The statistical analysis was conducted using a paired student's *t*‐test (*P < 0.05, **P < 0.01, ***P < 0.001).

To study whether IIS regulates glucose transport activity in *C. elegans*, we examined the activities of facilitated glucose transport and Na^+^‐dependent glucose transport in wt and IIS mutant animals by measuring the uptake activities of tritium‐labeled 2DG (^3^H‐2DG) in wt and mutant strains of *fgt‐1(tm3165)*,* daf‐2(e1370)*,* fgt‐1(tm3165); daf‐2(e1370)*,* age‐1(hx546)*,* akt‐1(mg306)*, and *daf‐16(mgDf50); daf‐2(e1370)* in the presence or absence of phloretin, a facilitated glucose transport inhibitor, or phlorizin, a Na^+^‐dependent glucose transport inhibitor (Fig. 2). The null function of FGT‐1 in *fgt‐1(tm3165)* mutant was confirmed in Fig. 1D. First, glucose uptake activities in *fgt‐1*,* daf‐2*,* fgt‐1; daf‐2*, and *age‐1* mutants were similar and approximately 40% lower than those in wt (*P* < 0.001). The *akt‐1* mutant also displayed lower glucose uptake than wt (*P* < 0.05) but higher uptake than the *fgt‐1*,* daf‐2*,* fgt‐1; daf‐2*, and *age‐1* mutants (*P* < 0.05). In addition, glucose uptake activity in *daf‐16; daf‐2* did not differ from that in wt (Fig. 2A). Second, phloretin significantly inhibited 2DG uptake in wt, *daf‐2*,* akt‐1*, and *daf‐16; daf‐2* (*P* < 0.05) but not in the *fgt‐1* and *fgt‐1; daf‐2* mutants. Phloretin tended to inhibit 2DG uptake in *age‐1* (*P* = 0.058) (Fig. 2B). Finally, phlorizin inhibited 2DG uptake in all of the tested strains (*P* < 0.05) (Fig. 2C).

### IIS regulates FGT‐1 expression

**Figure 3:**
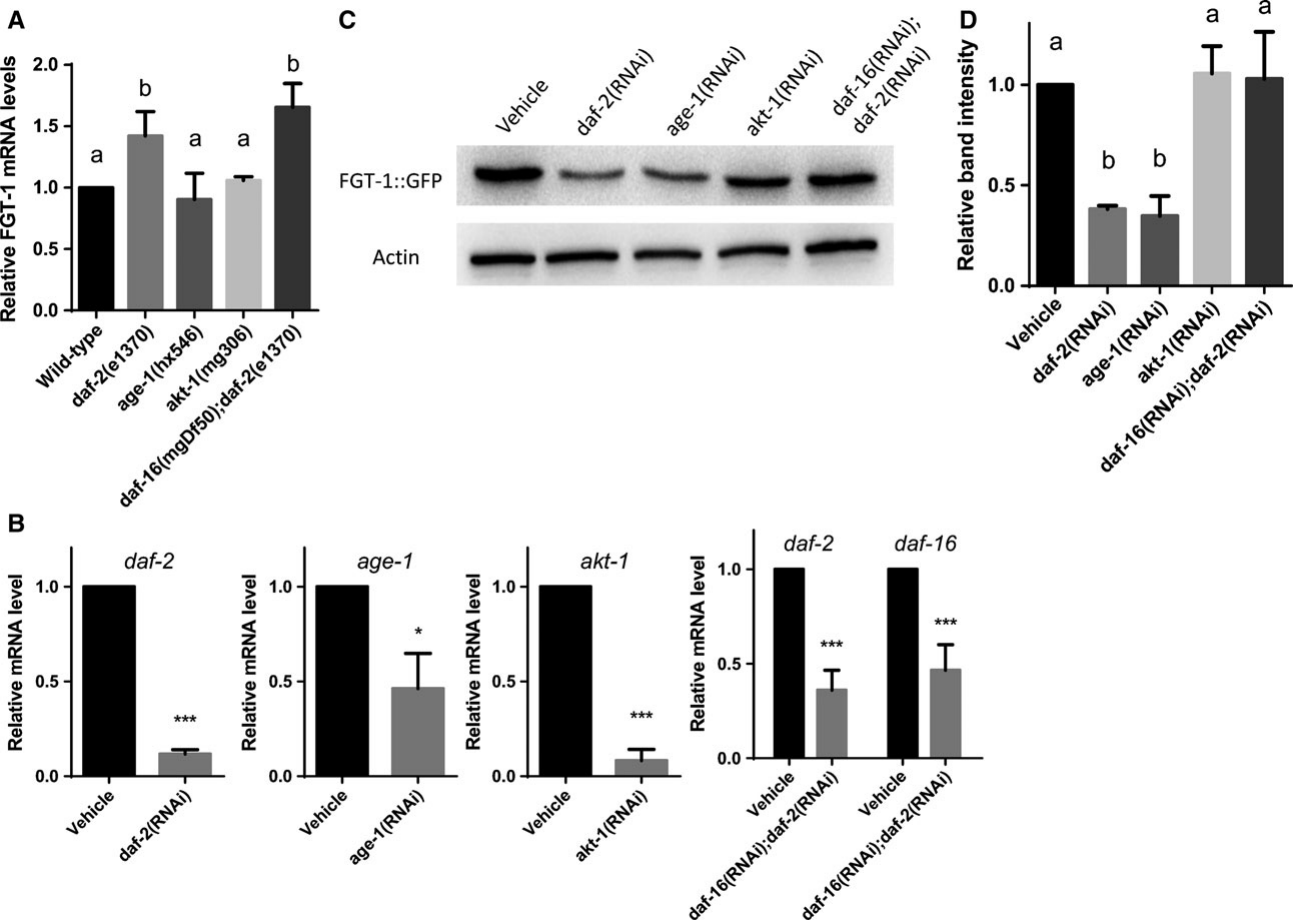
Effects of insulin/IGF‐like signaling on FGT‐1 mRNA and protein expression. (A) The mRNA levels of FGT‐1 in wild‐type and *daf‐2(e1370)*,* age‐1(hx546)*,* akt‐1(mg306)*, and *daf‐16(mgDf50); daf‐2* mutants were measured by real‐time reverse transcription PCR and normalized to the mRNA levels of CDC‐42 and PMP‐3. The mRNA level of FGT‐1 in wild‐type worms was set at 1. Error bars represent the SD (*N* = 3). The statistical analysis was conducted using one‐way ANOVA with Tukey's HSD test, and significant differences are indicated by the different alphabetical letters on each bar (*P* < 0.001). (B) Transgenic *C. elegans* expressing the FGT‐1::GFP fusion protein were fed individual RNAi bacteria of *daf‐2*,* age‐1*,* akt‐1*, or both *daf‐2* and *daf‐16* and analyzed for the relative mRNA levels of DAF‐2, AGE‐1, AKT‐1, or DAF‐16 by real‐time PCR. Error bars represent the SD (*N* = 3). The statistical analysis was conducted using the paired student's *t*‐test (*P < 0.05, ***P < 0.001). (C, D) Expression of the FGT‐1::GFP fusion protein in FGT‐1::GFP transgenic worms that were fed control RNAi bacteria (vehicle) or individual RNAi bacteria of *daf‐2*,* age‐1*,* akt‐1*, and both *daf‐2* and *daf‐16* were analyzed by western blot analysis with an antibody against GFP (C). Actin served as an internal control. The relative FGT‐1::GFP levels were quantitated (D). Error bars represent SD (*N* = 3). The statistical analysis was conducted by one‐way ANOVA with Tukey's HSD test, and significant differences are indicated by the different alphabetical letters on each bar (*P* < 0.001).

To study the mechanisms underlying the impaired facilitated glucose transport activity in the IIS mutants, the mRNA level of FGT‐1 was analyzed in wt and IIS mutant worms by RT‐qPCR. Although the FGT‐1 mRNA level remained unchanged in *age‐1(hx546)* and *akt‐1(mg306)* compared to wt, it was approximately 50% higher in the *daf‐2(e1370)* and *daf‐16(mgDf50); daf‐2(e1370)* mutants (*P* < 0.001) (Fig. 3A).

Due to the lack of FGT‐1 antibody, we analyzed the effect of IIS on FGT‐1 protein expression in the transgenic worm expressing the FGT‐1::GFP fusion protein using an antibody against GFP. The IIS in the transgenic worm was manipulated by feeding RNAi bacteria of *daf‐2*,* age‐1*,* akt‐1*, or both *daf‐16* and *daf‐2*. Real‐time RT‐qPCR confirmed the effectiveness of each individual bacteria‐mediated RNAi (Fig. 3B). Expression of the FGT‐1::GFP fusion protein in the transgenic worm was decreased by more than 50% by *daf‐2* and *age‐1* RNAi (*P* < 0.05) but not by *akt‐1* and *daf‐16; daf‐2* RNAi (Fig. 3C,D).

### The glycosylation gene OGA‐1, an O‐GlcNAcase of *C. elegans*, affects glucose uptake in animals

**Figure 4:**
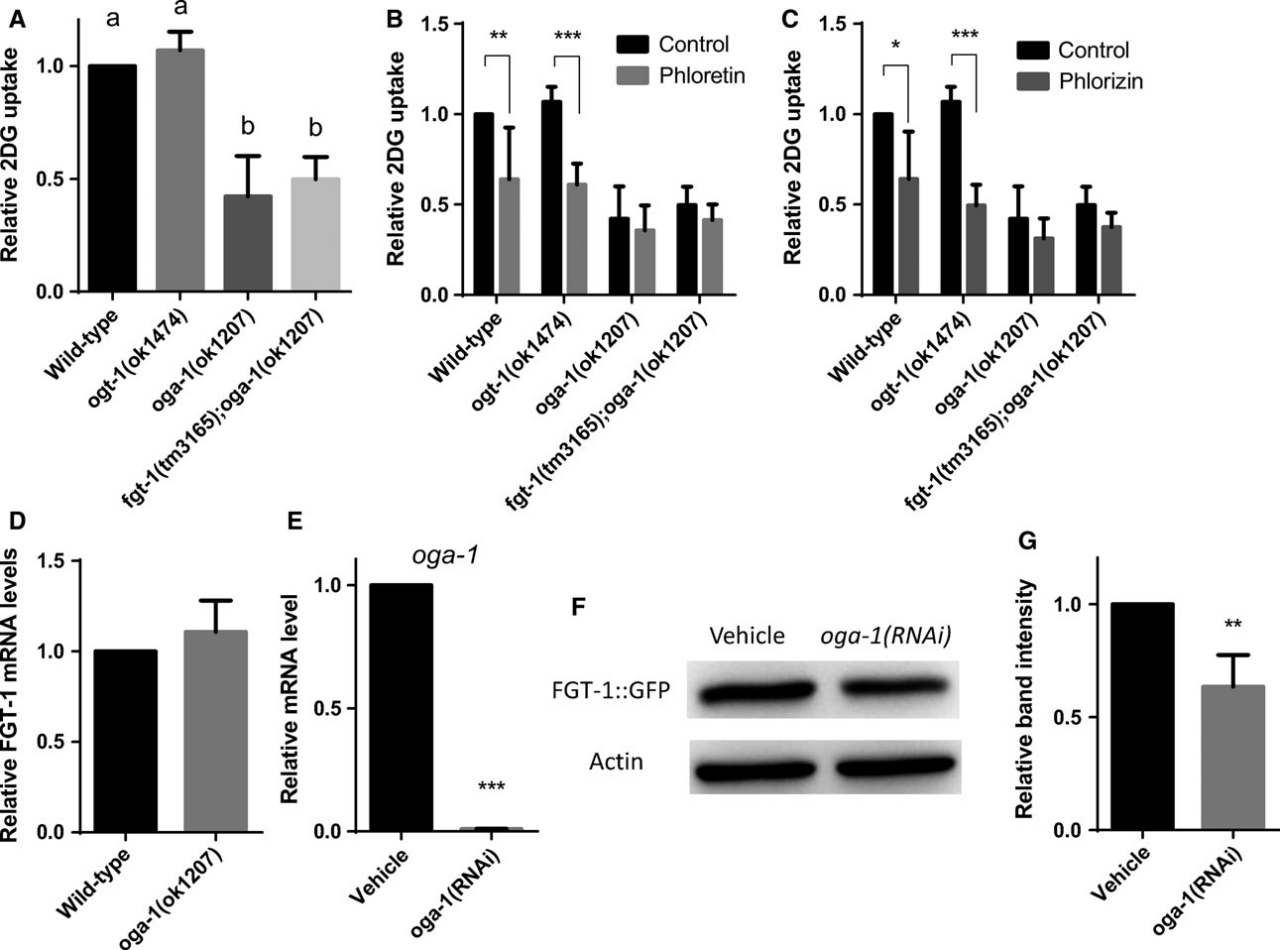
Effect of the mutation of glycosylation genes on glucose uptake and FGT‐1 expression. (A–C) The 2‐deoxy‐d‐glucose (2DG) uptake activity in wild‐type, *ogt‐1(ok1474)*,* oga‐1(ok1207)*, and *fgt‐1(tm3165); oga‐1(ok1207)* was analyzed in the presence of no inhibitor (A), phloretin (B), or phlorizin (C). Worms were incubated with tritium‐labeled 2DG (^3^H‐2DG) for 2 h and assessed for the uptake of 2DG. Uptake data of wild‐type worms (same as in Fig. 2B–D) was set at 1. Error bars represent the SD (*N* = 4). (D) mRNA levels of FGT‐1 in wild‐type and *oga‐1(ok1207)* were analyzed by real‐time reverse transcription PCR (RT‐qPCR) and normalized to the mRNA levels of CDC‐42 and PMP‐3. The mRNA level of FGT‐1 in wild‐type worms was set at 1. Error bars represent the SD (*N* = 3). (E) Transgenic *C. elegans* expressing the FGT‐1::GFP fusion protein were fed RNAi bacteria of *oga‐1* or the vector control (vehicle) and analyzed for the mRNA level of OGA‐1 by RT‐qPCR. Error bars represent the SD (*N* = 3). (F, G) The expression of the FGT‐1::GFP fusion protein in FGT‐1::GFP transgenic worms fed either control RNAi bacteria (vehicle) or RNAi bacteria of *oga‐1* were analyzed by western blot analysis using an antibody against GFP (F). ACT‐1 served as an internal control for normalization. The relative FGT‐1::GFP levels were quantitated (G). Error bars represent the SD (*N* = 3). In A, the statistical analysis was conducted using one‐way ANOVA with Tukey's HSD test, and significant differences are indicated by the different alphabetical letters on each bar (*P* < 0.001). In B–E and G, the statistical analysis was performed using the paired student's *t*‐test (*P < 0.05, **P < 0.01, ***P < 0.001).

In humans, *N*‐ and *O*‐linked glycosylation modifications are known to regulate the function of GLUT1 18, 19. The amino acid sequence of FGT‐1 does not contain any predicted *N*‐glycosylation sites (NetNGlyc 1.0 Server) 20 but have potential *O*‐linked glycosylation sites (NetOGlyc 4.0 Server). In *C. elegans*, OGT‐1 and OGA‐1 encode the sole *O*‐GlcNAc transferase and *O*‐GlcNAcase, respectively 21. To study the potential regulation of these glycosylation genes in glucose uptake in *C. elegans*, we first assessed 2DG uptake activity in the *ogt‐1(ok1474)* and *oga‐1(ok1207)* mutant strains. Although the *ogt‐1* mutant showed the same level of 2DG uptake activity as wt, the transport activity was sharply decreased in the *oga‐1* mutant (*P* < 0.001) (Fig. 4A). In addition, both phloretin and phlorizin inhibited 2DG uptake in the *ogt‐1* mutant (*P* < 0.001) but not in the *oga‐1* mutant (Fig. 4B,C), indicating that the *oga‐1* mutant strain has impairments in both facilitated glucose transport and Na^+^‐dependent transport activities. The double mutant of *fgt‐1(tm3165); oga‐1(ok1207)* showed the same level of uptake activity as the *oga‐1(ok1207)* mutant, which suggested that the impaired 2DG uptake in the *oga‐1* mutant resulted from the impaired function of FGT‐1. To examine this possibility, the mRNA and protein levels of FGT‐1 were analyzed in *oga‐1* mutant or knockdown animals. There were no differences in FGT‐1 mRNA levels between wt and *oga‐1* mutant strains, as measured by real‐time RT‐qPCR (Fig. 4D). However, OGA‐1 knockdown by RNAi (Fig. 4E) reduced the expression of the FGT‐1::GFP fusion protein by 37% (*P* < 0.001) in FGT‐1::GFP transgenic worms (Fig. 4F,G). Taken together, our data demonstrated that OGA‐1 might be involved in the regulation of expression and function of FGT‐1 in *C. elegans*.

## Discussion

### FGT‐1 is the sole functional GLUT homolog, and the existence of Na^+^‐dependent glucose transport in *C. elegans*

In our previous study 12, we investigated the glucose transport activity of nine candidates of GLUT homologs in *C. elegans* based on their sequence homologies to GLUTs and found that FGT‐1 was the only protein with transport activity for 2DG in *Xenopus* oocytes. Feng *et al*. 17 conducted a similar study and reached the same conclusion. In the present study, we provided further evidence supporting that FGT‐1 is the only functional GLUT homolog in *C. elegans*: (a) In contrast to wt, the impaired 2DG uptake in the FGT‐1 null function mutant *fgt‐1(tm3165)* could not be further inhibited by phloretin, a GLUT inhibitor 22, whereas it could be further inhibited by phlorizin, a Na^+^/glucose cotransporter (SGLT) inhibitor 23 (Fig. 2); (b) Under the control of the 5 kb *fgt‐1* promoter, FGT‐1 was ubiquitously expressed in *C. elegans* (Fig. 1) 12, suggesting that FGT‐1 may be ubiquitously involved in glucose transport in the tissues and cells of *C. elegans*. In addition, the current study showed that two FGT‐1‐splicing isoforms, FGT‐1A and ‐1B, likely have the same or similar tissue distributions and glucose transport activity in *C. elegans* (Fig. 1). Furthermore, we also demonstrated the presence of Na^+^‐dependent glucose transport in *C. elegans* based on the inhibition of 2DG uptake activity by phlorizin (Fig. 2). However, blastp searches using protein sequences of human SGLTs against the *C. elegans* database (www.wormbase.org) did not reveal any proteins with significant sequence homology to SGLTs. Thus, the transporters responsible for Na^+^‐dependent glucose transport in *C. elegans* remain to be identified.

### IIS mutants have impaired FGT‐1‐mediated glucose transport

Consistent with the findings of Feng *et al*. 17, our study showed the decreased glucose uptake and FGT‐1 activity in IIS mutants. Our study further showed that in *daf‐2* mutant animals, 2DG uptake activity was decreased to a level similar to that in *fgt‐1* mutant animals because the activity did not further decrease in the *fgt‐1; daf‐2* double mutant and could be inhibited only slightly by phloretin. These results indicated that FGT‐1‐mediated glucose uptake was almost completely impaired in the *daf‐2* mutant. In addition, our data showed that the impaired function of FGT‐1 in the *daf‐2* mutant was dependent on the DAF‐16 transcription factor because the DAF‐16 mutation in the *daf‐2* mutant completely recovered the decreased 2DG uptake (Fig. 2B). Furthermore, our study showed that 2DG uptake was also significantly impaired in other IIS mutants, including *age‐1* and *akt‐1*. The uptake observed in the *age‐1* mutant was similar to that in the *daf‐2* mutant, whereas the uptake in the *akt‐1* mutant was between that of the wt and the *daf‐2* and *age‐1* mutants (Fig. 2B). In addition, FGT‐1 protein level was dramatically decreased in AGE‐1 RNAi, but not in AKT‐1 RNAi (Fig. 3D). These observations suggest that AGE‐1 may mediate the stimulation of FGT‐1 function by DAF‐2, whereas AKT‐1 was only partially involved in this process. In the IIS cascade in *C. elegans*, there are at least two additional signaling molecules downstream of AGE‐1: AKT‐2 and SGK‐1 24, 25. These signaling pathways may also cooperatively function with AKT‐1 to mediate DAF‐2 regulation of FGT‐1 function. Because 2DG uptake could be inhibited by phlorizin in all of the tested strains to similar degrees (Fig. 2C), Na^+^‐dependent glucose transport may not be regulated by IIS in *C. elegans*, which is similar to the phenomenon observed in mammals 26, 27.

### Regulation of FGT‐1 expression by IIS

To study the mechanism underlying the serious defect in FGT‐1‐mediated glucose uptake in IIS mutants, we first studied the expression of FGT‐1 in IIS mutants. The mRNA expression of FGT‐1 was not decreased in IIS mutants, but was even increased in *daf‐2* and *daf‐16; daf‐2* mutants (Fig. 3A), excluding the transcriptional inhibition of FGT‐1 expression by IIS. However, the protein level of FGT‐1 was substantially lower in DAF‐2 and AGE‐1 knockdown worms compared to control worms, and the decreased FGT‐1 protein level in *daf‐2* mutant is in a *daf‐16*‐dependent manner (Fig. 3C,D). This decreased protein expression should at least partially contribute to the impaired FGT‐1‐mediated glucose uptake in the *daf‐2* and *age‐1* mutants. At present, it is not known whether this decreased protein expression of FGT‐1 was due to a decrease in mRNA stability or translational efficiency, or an increase in protein degradation. It is noteworthy again that the FGT‐1 protein level did not decrease in the AKT‐1 knockdown animals (Fig. 3C, D), indicating that the depressed FGT‐1 protein expression was not the only mechanism responsible for the impaired glucose uptake in the IIS mutants.

### Post‐translational regulation of FGT‐1

Additional evidence that IIS does not regulate the function of FGT‐1 solely by reducing FGT‐1 protein expression in *C. elegans* is provided by our observation that FGT‐1‐mediated glucose uptake in the *daf‐2* mutant was almost completely abrogated (Fig. 2B), whereas the protein level of FGT‐1 was only reduced to 40% (Fig. 3D). Other mechanisms could account to the function of the remaining 40% of FGT‐1 protein. One possible mechanism is the regulation of the FGT‐1 subcellular localization by IIS, however, our preliminary Wormbase search showed no conserved homologs in the *C. elegans* genome of some of the key molecules involved in GLUT4 translocation such as AS160 28, 29.

Another potential mechanism is the regulation of FGT‐1 glycosylation by IIS. In mammals, the function of GLUT1, a GLUT that is systemically expressed throughout the body and is responsible for basic cellular glucose uptake, is fine‐tuned by tissue‐specific glycosylation modifications 18, 19, 30, 31. In the present study, we could only examine the effects of the mutation of genes involved in glycosylation in *C. elegans* on the function of FGT‐1. Because FGT‐1 does not have any predicted *N*‐linked glycosylation sites, we investigated the effects of mutation of the *O*‐linked glycosylation enzymes OGT‐1 and OGA‐1. Intriguingly, OGA‐1, a homolog of *O*‐GlcNAcase, was found to be essential for the function of FGT‐1 as well as Na^+^‐dependent glucose uptake (Fig. 4). This observation is consistent with the finding of Rahman *et al*. 21 that DAF‐16 is required for the longevity of *oga‐1* mutant because this study showed that IIS regulation of FGT‐1 function is DAF‐16 dependent. However, the total defect in FGT‐1‐mediated glucose uptake in the *oga‐1* mutant could not be solely due to the approximately 30% decrease in FGT‐1 protein and is likely related to the modification of the FGT‐1 glycosylation status. Surprisingly, the mutation of OGT‐1, an *O*‐GlcNAc transferase, showed no effect on glucose uptake in *C. elegans* (Fig. 4). This finding may be explained by the observation that *O*‐linked glycosylation of FGT‐1 is not dependent on the function of OGT‐1, but on other factors. Nevertheless, further studies are required to investigate FGT‐1 glycosylation and its potential regulation by IIS.

## Conclusions

In this study, we demonstrated that in *C. elegans*, FGT‐1‐mediated facilitated glucose uptake is severely defective in IIS mutants and that DAF‐2, AGE‐1, and AKT‐1 are involved in the control of FGT‐1 function by regulating its protein expression and by other unidentified mechanisms. The results of our study are important in at least two research fronts: (a) We establish *C. elegans* as a potential unique model to study the mechanisms of insulin regulation of glucose transport because of its relative ease of genetic manipulation and (b) *C. elegans* may be used as a drug‐screening model to search for compounds that stimulate glucose uptake in patients with diabetes.

## Author contributions

SK designed and performed experiments and wrote the manuscript; ADM provided equipment and revised the manuscript; F‐QZ designed experiments and revised the manuscript.

## Supporting information


**Data S1.** Materials and methods.
**Table S1.** List of primer sets.
