# Supplementary material for: FGT‐1‐mediated glucose uptake is defective in insulin/IGF‐like signaling mutants in Caenorhabditis elegans
Source: FEBS Open Bio. 2016 Apr 21;6(6):576–85. doi: 10.1002/2211-5463.12068 (PMC4887973; doi:10.1002/2211-5463.12068)
Supplement: Supplementary file 1 — Data S1. Materials and methods. Table S1. List of primer sets. [file FEB4-6-576-s001.docx]

# Supporting Materials and Methods

## *C. elegans* strains

*C. elegans* strains wild-type (N2), *daf-2(e1370), age-1(hx546), akt-1(mg306), daf-16(mgDf50), daf-16(mgDf50); daf-2(e1370), oga-1(ok1207)*, and *ogt-1(ok1474)* were obtained from the Caenorhabditis Genetics Center (University of Minnesota), and *fgt-1(tm3165)* was provided by the National BioResource Project (Tokyo Women’s Medical University School of Medicine). The double mutant of *fgt-1(tm3165); daf-2(e1370)* was constructed by crossing *fgt-1(tm3165)* and *daf-2(e1370)*, and the *fgt-1(tm3165)* and *daf-2(e1370)* alleles were confirmed by allele-specific PCR using the primer sets ‘tm3165chk’ and ‘e1370chk’, respectively [1]. The double mutant of *fgt-1(tm3165); oga-1(ok1207)* was constructed by crossing *fgt-1(tm3165)* and *oga-1(ok1207)*, in which the *fgt-1(tm3165)* allele was confirmed by allele-specific PCR with the primer set ‘tm3165chk’, and the *oga-1(ok1207)* allele was verified with two sets of primers: ‘ok1207chk’ and ‘ok1207WT’.

Transgenic worms were generated as described previously [2]. The *rol-6d* pRF4 (obtained from M. Koelle, Yale University) was used as a co-injection marker [3,4]. To generate the transgenic worm harboring the FGT-1B::GFP expression vector under the 2 kb FGT-1B promoter, the pPD-fgt1bg2k plasmid was microinjected into the wt worm. A transgenic line expressing FGT-1A::GFP under the 2 kb FGT-1A promoter was constructed in our previous study [5]. To observe FGT-1A::GFP localization under the 5 kb FGT-1A promoter, the pPD-fgt1g5k plasmid was microinjected into wt or *daf-2(e1370)* animals. All of the microinjected DNA solutions were prepared at a final concentration of 100 μg/mL. Transgenic animals bearing the pRF4 marker construct were selected based on the roller phenotype, and other strains were isolated by GFP reporter fluorescence observed under a confocal laser microscope (Zeiss LSM 510 META Laser Scanning Microscope). All of the primer sequences used herein are listed in Table S1.

## Plasmid constructions

All of the PCR reactions used for the plasmid constructions were conducted using high-fidelity Platinum *Pfx* DNA polymerase (Life technologies) unless otherwise specified.

To construct the *C. elegans* expression vector of FGT-1B::GFP under the control of the 2 kb *fgt-1b* 5’ upstream promoter sequence, FGT-1B cDNA was amplified from the cDNA library of the wt worm with the primer set ‘fgt1bcDNA’, and the amplicon was inserted into the *Sal* I site of pPD95.79 (Addgene plasmids #1496). The 2 kb 5’ upstream region of the *fgt-1b* gene was amplified from the genomic DNA of wt with the primer pair ‘fgt1bup2k’, and the amplicon was inserted into the *Sph* I and *Sal* I sites of pPD95.79 harboring the FGT-1B cDNA to form pPD-fgt1bg2k. For the expression vector of FGT-1A::GFP containing 5 kb of the *fgt-1a* 5’ upstream promoter sequence, the FGT-1A cDNA was amplified from the cDNA library of wt with the primer set ‘fgt1acDNA’, and the amplicon was inserted into the *Sma* I and *Sal* I sites of pPD95.79. The 5 kb 5’ upstream sequence of the *fgt-1a* gene was amplified from the genomic DNA of wt with the primer pair ‘fgt1bup5k’ and the Qiagen LongRange PCR Kit (Qiagen). The amplicon was inserted into the *Sph* I and *Sal* I sites of pPD95.79 harboring FGT-1A cDNA to form pPD-fgt1b5k.

To construct the *Xenopus* oocyte expression plasmid, the full-length cDNAs of FGT-1B and mutated FGT-1A and -1B were amplified from the cDNA library of wt or *fgt-1(tm3165)* worms, respectively. The primer set ‘fgt1bcDNAX’ was used to clone the wt and mutated FGT-1B cDNAs, and ‘fgt1acDNAX’ was used for the mutated FGT-1A cDNA. The cDNA amplicons of FGT-1B and mutated FGT-1A and -1B were inserted into the *Bgl* I site of pSP64T (obtained from Gwyn Gould, University of Glasgow) [6] to form pSP-fgt1b, pSP-fgt1atm, and pSP-fgt1btm, respectively.

# References

1 Zhang P (2011) PCR-RFLP Genotyping of Point Mutations in Caenorhabditis elegans. *Bio-protocol* **1**, e128.

2 Takashima Y, Kitaoka S, Bando T & Kagawa H (2012) Expression profiles and unc-27 mutation rescue of the striated muscle type troponin I isoform-3 in Caenorhabditis elegans. *Genes Genet Syst* **87**, 243–251.

3 Kramer JM, French RP, Park EC & Johnson JJ (1990) The Caenorhabditis elegans rol-6 gene, which interacts with the sqt-1 collagen gene to determine organismal morphology, encodes a collagen. *Mol Cell Biol* **10**, 2081–2089.

4 Mello C & Fire A (1995) DNA transformation. *Methods Cell Biol* **48**, 451–482.

5 Kitaoka S, Morielli AD & Zhao FQ (2013) FGT-1 Is a Mammalian GLUT2-Like Facilitative Glucose Transporter in Caenorhabditis elegans Whose Malfunction Induces Fat Accumulation in Intestinal Cells. *PLoS One* **8**, e68475.

6 Gould GW, Thomas HM, Jess TJ & Bell GI (1991) Expression of human glucose transporters in Xenopus oocytes: kinetic characterization and substrate specificities of the erythrocyte, liver, and brain isoforms. *Biochemistry* **30**, 5139–5145.

# Supporting Tables

## Table S1. List of primer sets

| Name | Forward primer | Reverse primer |
| --- | --- | --- |
| e1370chk | CGGGATGAGACTGTCAAGATTGGAGATTTCGG | CAACACCTCATCATTACTCAAACCAATCCATG |
| tm3165chk | GCGGGGGTTCAGCTGTGTAA | TATGATACGGAGTTTCGCCA |
| ok1207chk | GCAAACAGAAAGGTGAGCTAG | AACTCAGAAGGCACAGGCTC |
| ok1207WT | ACGAGCGGTTCCGACGTTAG | TTCCGACGCATTACACTTCC |
| FGT1q | TCCAGGAGGGCTTATCAC | AATACGGATACTGCGACGGAC |
| DAF2q | GTGGCGTGAGAATGAAGTGAG | GGAATTTCGTAGAATCCGTTG |
| AGE1q | CGGAAAGACCAAACTTGGGATC | CGTAGGCTTCGACGCATAACG |
| AKT1q | CAAAGCCTAAGGAAGGACAACC | CATGAATCCAACGCTGACGAAC |
| DAF16q | TCGTCGTCTCGTGTTTCTCCA | TTCCATAGGCACCCGGTAGTG |
| OGA1q | ACATTTCCCGATTAAGTATTGACTGTG | AGATGCTTGCGCTGTTCTGG |
| CDC42q | CTGCTGGACAGGAAGATTACG | CTCGGACATTCTCGAATGAAG |
| PMP3q | GTTCCCGTGTTCATCACTCAT | ACACCGTCGAGAAGCTGTAGA |
| fgt1bcDNA | AAGTCGACGAAGAAAATTCATTTAAAATAATGTCGGA | CTTCCTCTTCTCGAATTCG |
| fgt1b2kbup | CATGCATGCATTTGGAGCGAATCAGGC | AAGTCGACGAAATAAATATATATTTTCTAGATTTAGACG |
| fgt1acDNA | AAGTCGACAGAAATGGGTGTCAACGACCATG | CTTCCTCTTCTCGAATTCG |
| fgt1b5kbup | CATGCATGCAGACGGCTGAAAGTGCGCTCCAG | AAGTCGACGAAATAAATATATATTTTCTAGATTTAGACG |
| fgt1bcDNAX | TACTGATCAGAAGAAAATTCATTTAAAATAATGTCGGA | TACTGATCAAACCTATACGTTTCGCAGTG |
| fgt1acDNAX | TACTGATCAAATGGGTGTCAACGACCATG | TACTGATCAAACCTATACGTTTCGCAGTG |
